# Bowler's Thumb: Case Report and Review of the Literature

**Published:** 2015-10-24

**Authors:** Jordan N. Halsey, Paul J. Therattil, Stephen L. Viviano, Earl J. Fleegler, Edward S. Lee

**Affiliations:** ^a^Division of Plastic and Reconstructive Surgery, Department of Surgery, Rutgers New Jersey Medical School, Newark; ^b^Department of Veterans Affairs New Jersey Health Care System, East Orange

**Keywords:** bowler’s neuroma, bowler’s thumb, digital neuroma, nerve wrap, thumb neuroma

## Abstract

**Objective:** Traumatic neuropathy of the ulnar digital nerve of the thumb occurs in patients who undergo chronic frictional irritation of the nerve. The condition is aptly termed bowler's thumb, as it commonly afflicts patients who bowl and keep their thumb in the ball for an extended period of time. It is a pathology that rarely appears in the literature and for which standard treatment is unclear. **Methods:** A 68-year-old man with a history of avid bowling presented with a chief complaint of left thumb numbness and tingling for several months. Physical examination demonstrated a small tender mass along the ulnar surface of the left thumb with a positive Tinel's sign and diminished 2-point discrimination distal to the mass. The patient's symptoms did not improve with conservative measures. Here, we describe his surgical treatment and review the current literature. **Results:** Our patient underwent neurolysis of the ulnar digital nerve of the thumb, with placement of a porcine extracellular matrix nerve wrap. The patient reported improvement of symptoms within 2 weeks and was able to return to his previous level of activity. In the literature, treatment has included from conservative measures such as pressure relief and splinting to surgical intervention including neurolysis, transposition, or neurectomy with nerve grafting. **Conclusions:** We present neurolysis for treating bowler's thumb as a viable option that may relieve symptoms rapidly while obviating need for adductor transection. Porcine extracellular matrix nerve wrap can be placed in an attempt to prevent recurrence of neuroma, but long-term follow-up is necessary.

Bowler's thumb is a traumatic neuropathy in which the ulnar digital nerve of the thumb undergoes chronic frictional irritation that becomes symptomatic. The condition is aptly termed bowler's thumb, as it commonly afflicts tenpin bowlers who keep their thumb in the ball for an extended period of time to create spin following release. Patients typically present with localized pain and tenderness at the first web space, numbness, and/or paresthesias that extend distally from the first web space along the medial thumb surface. On physical examination, a small mass may be palpable and will often be tender with hyperesthesia in the surrounding areas.^1^ Increased 2-point discrimination is often observed.^2^

Case reports have documented a similar constellation of symptoms not only in bowlers but also in jewelers, cherry pitters, baseball players, and massage therapists.^1^,^3^^-^5 The pathophysiology remains the same in all these groups—chronic tension and irritation along the nerve cause traction neuritis with or without neuroma formation. On histologic analysis, previous case series have demonstrated scar tissue enveloping the nerve or atrophy of the nerve fascicles with invading fibrosis between fascicles when nerve resection was performed.^6^

Treatment of bowler's thumb is typically conservative, with recommended rest, relief of pressure, and avoidance of the inciting activity.^7^ When symptoms remain refractory to conservative measures, surgery is the alternative option. Several techniques have been described including neurolysis, neurectomy, and transposition of the ulnar digital nerve.^2^ Here, we present the case of a patient with a remote but avid bowling history who had symptoms consistent with bowler's thumb, his treatment, and subsequent outcome. In addition, we review the current literature.

## CASE REPORT

Our patient was a 68-year-old, left-hand-dominant man who presented with a chief complaint of left thumb numbness with a palpable mass along the flexor surface of his thumb. The patient noted tingling in his left hand, mainly at night, for the last 3 years. He had difficulty performing simple tasks of daily living with the left hand due to thumb numbness. The patient was, significantly, an avid bowler in the past and reported weakened grip while bowling since the onset of his symptoms. At the time of presentation, the mass had been present for 2 months and was tender to palpation. The patient denied any history of hand trauma or previously diagnosed pathologic nerve compression. His medical history included gastroesophageal reflux disease, chronic obstructive pulmonary disease, and mild arthritis. He denied any history of diabetes or having hypothyroidism.

Pertinent positives on patient's physical examination included a positive Tinel's sign over the left thumb mass and also at the carpal tunnel bilaterally. The patient had a positive carpal compression test on the left. He also had decreased range of motion at the neck but a negative Spurling's maneuver. Static 2-point discrimination was at least 5 mm at all digits except at the ulnar digital nerve of the left thumb, which was greater than 15 mm. There were no motor or vascular abnormalities.

The patient underwent electrodiagnostic testing, which was negative for any compressive neuropathy or cervical radiculopathy. Magnetic resonance imaging of his left hand demonstrated no definite abnormal signal or lesion at the region corresponding to the palpable mass. Once the diagnosis of bowler's thumb was made, the patient underwent a trial of splinting for 1 month with no change and then opted for surgical intervention.

**Figure 1:**
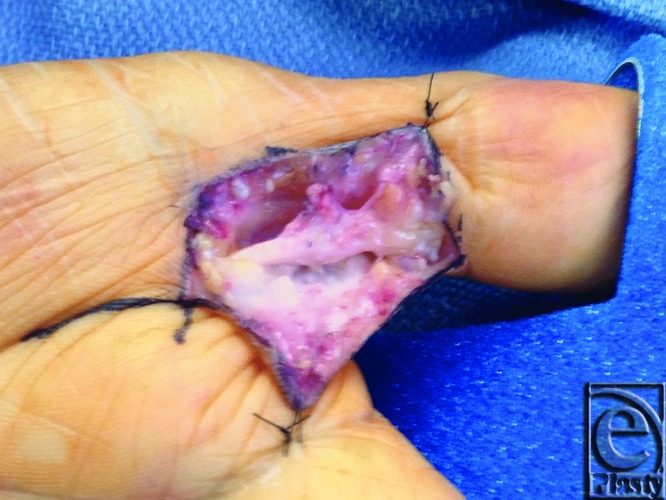
Intraoperative view of extensive scar and fibrosis surrounding the left ulnar digital nerve of the thumb.

**Figure 2:**
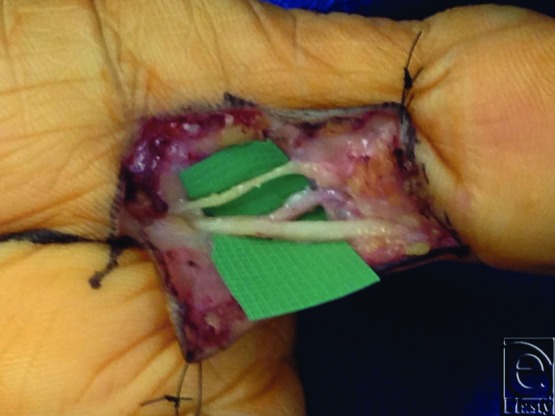
Intraoperative view of the left ulnar digital nerve of the thumb after neurolysis.

**Figure 3:**
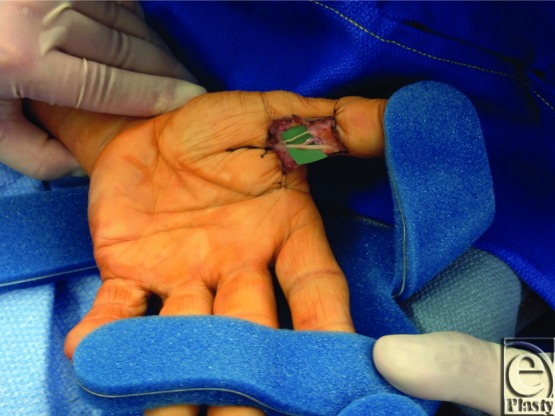
Intraoperative view of the left ulnar digital nerve of the thumb after neurolysis, with view of the entire left hand.

**Figure 4:**
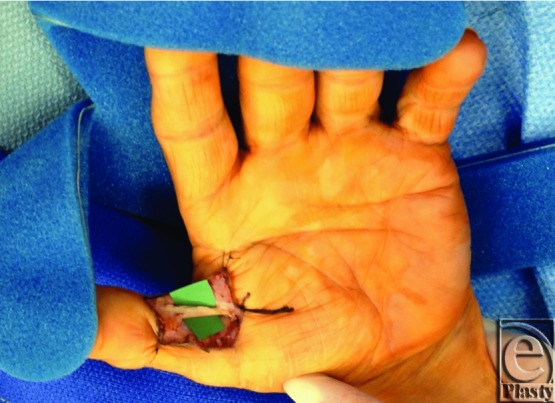
Intraoperative view of the left ulnar digital nerve of the thumb with AxoGuard Nerve Protector in place.

At time of operation, a Bruner incision was marked out over the proximal aspect of the ulnar side of the thumb with an extension into the palm. The ulnar digital nerve of the left thumb was exposed, revealing extensive scar tissue and fibrosis surrounding approximately 3 cm of the nerve (Fig 1). While freeing the nerve from the surrounding fibrosis, branches of the ulnar digital nerve were encountered and preserved. Neurolysis of the ulnar digital nerve was completed (Figs 2 and 3) and the nerve was then wrapped with an AxoGuard Nerve Protector (AxoGen, Inc, Alachua, Fla) cut to size (Fig 4). The nerve protector was placed in 2 different locations, both proximally and distally at the nerve, and loosely sewn into place with 8-0 interrupted nylon sutures. The incision was then closed with 4-0 nylon interrupted horizontal mattress sutures.

The patient was seen 6 days postoperatively. At this time, the patient already reported alleviation of his pain and numbness. On physical examination, the patient's 2-point discrimination was found to be 5 mm at the ulnar side of the thumb. At subsequent visits, the patient continued to report resolution of his symptoms and ability to return to his regular activities. Tinel's sign at the thumb web space after the procedure was no longer present.

## DISCUSSION

Bowler's thumb has been described in the literature previously, although case reports are limited and treatment options have varied widely. We performed a PubMed literature search with terms including “bowler's thumb,” “bowler's neuroma,” “cherry pitter's thumb,” “digital neuropathy of the thumb,” “digital neuroma of the thumb,” and “jeweler's thumb” to determine the various presentations of the disorder, workup, and treatments that have been attempted.

Although at first glance bowler's thumb appears to be a narrow diagnosis, it actually encompasses a number of entities. As mentioned earlier, compression neuropathy of the ulnar digital nerve of the thumb can occur from a number of inciting factors beyond bowling. Interestingly, a similar condition can occur acutely, with some patients developing symptoms with a single night of bowling. The difference between acute neuropathy of the ulnar digital nerve of the thumb and the originally described bowler's thumb is that the former is thought to be a neurapraxia or axonotmesis that will resolve spontaneously within months. Furthermore, exploration of suspected bowler's thumbs has revealed multiple possible pathologies, including neuroma, or only surrounding fibrosis and scar with no neuroma.^8^ This is significant, as it may change the planned surgical treatment.

Workup of bowler's thumb may include nerve conduction studies, which may be normal, or may demonstrate decreased median sensory nerve action potential amplitude. Latencies generally remain normal, however. If bowler's thumb is not on the initial differential diagnosis, magnetic resonance imaging might be performed to evaluate the unknown thumb mass, which may not demonstrate any abnormality, as in our patient.

Once the diagnosis of bowler's thumb is made, patients should undergo an initial period of conservative management if possible. In prior case series, about half of affected patients appeared to show improvement of their symptoms with conservative management.^6^ Patients who stopped bowling tended to have marked improvement of their symptoms when compared with those patients who continued to bowl. For those who continue to bowl, neoprene sleeves can be used to ease compression of the nerve and splinting can be utilized to decrease inflammation and protect the thumb from repetitive trauma.^5^^-^7 Other conservative measures described specifically for bowlers include changing the weight of the bowling ball used or the distance, size, and/or slope of the thumb hole. In previous case series, all those who returned to bowling required some change in grip or thumb-hole placement.^9^ In professional bowlers or others who cannot afford to undergo a period of conservative treatment, however, surgical exploration may be one of the initial steps. Similarly, for those who fail conservative treatment, as in our patient, surgery may be required to alleviate symptoms.

Most patients who undergo surgical intervention for bowler's thumb are able to return to the sport within 2 years (50%–100% success rate).^6^ Surgical options described include neurolysis, neuroma resection with or without nerve graft, transposition of the ulnar digital nerve, and transposition deep to the adductor pollicis.^2^ Some describe simple neurolysis to be sufficient in alleviating symptoms, but the concern with neurolysis alone is the possible risk of neuroma recurrence, especially in avid bowlers who continue to perform activities causing direct trauma to the ulnar digital nerve. In our patient, we utilized the AxoGuard Nerve Protector to assist in guarding the nerve from trauma, with favorable results observed thus far during follow-up visits. This particular nerve wrap is a porcine submucosa-derived extracellular matrix. The goal of the wrap is to allow the body to form a new epineurium without intervening fibrosis and scar tissue. Prior studies using murine models have demonstrated that the use of vein wraps around nerve compression sites allows for less nerve degeneration, less scar, and less nerve latency than nonwrapped nerves.^9^ Nerve wraps attempt to mimic this effect without the sacrifice of additional donor sites. Although nerve wraps have been used for many years for nerve injuries, the literature on them is lacking. Thus far, data are available for the use in recurrent cubital tunnel syndrome.^10^ Nerve wraps appear to be safe, but comparison with surgical procedures not utilizing nerve wraps is not yet available.

Transposition of the ulnar digital nerve of the thumb provides additional protection, as the nerve is no longer subject to direct traumatic forces; it is protected by subcutaneous tissue, or muscle, in its new location. Transposition of the ulnar digital nerve was first described by Dunham et al.^7^ They utilized 7-0 silk tacking sutures to attach the subcutaneous tissue to the epineural sheath to pull the ulnar digital nerve more medially and away from pressure points. Both patients in the series had favorable results. Another transposition procedure was described by Belsky and Millender,^4^ which involved moving the ulnar digital nerve dorsal to the adductor pollicis. This required division of the adductor, transposition of the nerve dorsal to the muscle, and subsequent repair of the adductor. Swanson et al^2^ described a modification of this procedure with the use of a bone anchor rather than sutures to reattach the adductor.

The literature regarding bowler's thumb is relatively sparse and thus comparing the efficacy of treatment options for bowler's thumb is difficult at this point. What is apparent is that conservative management will work for some proportion of patients. Furthermore, outcomes for all described procedures have been mostly favorable, although recurrences have been observed in patients who underwent neurolysis alone. In addition, those who undergo neurectomy will have some degree of sensory loss. Therefore, we propose an algorithm for the treatment of bowler's thumb. First, conservative treatment should be undertaken with the previously described measures. The optimal time period for this is unknown. If conservative treatment fails, operative exploration should be performed. For patients who need to continue activities that will exacerbate the neuritis, submuscular transposition should be strongly considered. For all others, intraoperative evaluation will dictate management. The ulnar digital nerve of the thumb should be dissected free from scar. If no obvious neuroma is present, a nerve wrap may be placed in an attempt to prevent recurrence. If neuroma is present, neurectomy with nerve grafting might be considered.
